# Optimization of orbital retraction during endoscopic transorbital approach via quantitative measurement of the intraocular pressure – [SevEN 006]

**DOI:** 10.1186/s12886-021-01834-5

**Published:** 2021-02-08

**Authors:** Woohyun Kim, Ju Hyung Moon, Eui Hyun Kim, Chang-Ki Hong, Jisang Han, Je Beom Hong

**Affiliations:** 1 Department of Neurosurgery, Gangnam Severance Hospital, Yonsei University College of Medicine, Seoul, Korea; 2 Department of Neurosurgery, Severance Hospital, Yonsei University College of Medicine, Seoul, Korea; 3 Department of Ophthalmology, Kangbuk Samsung Hospital, Sungkyunkwan University School of Medicine, Seoul, Korea; 4 Department of Neurosurgery, Kangbuk Samsung Hospital, Sungkyunkwan University School of Medicine, 29 Saemunan-ro, Jongno-gu, Seoul, 03181 Korea

**Keywords:** Intraocular pressure, Intraorbital pressure, Orbital compression, Transorbital approach

## Abstract

**Background:**

Increased use of the transorbital approach (TOA) warrants greater understanding of the risk of increased intraocular pressure (IOP) and intraorbital pressure (IORP) due to orbital compression. We aimed to investigate the changes in IOP and IORP in response to orbital retraction in TOA and establish a method for the continuous measurement of intraoperative IORP.

**Methods:**

We assessed nine patients who underwent TOA surgery from January 2017 to December 2019, in addition to five cadavers. IORP and IOP were measured using a cannula needle monitor, tonometer, cuff manometer, and micro strain gauge monitor.

**Results:**

In all nine clinical cases and five cadavers, increased physical compression of the orbit increased the IOP and IORP in a curvilinear pattern. In clinical cases, when the orbit was compressed 1.5 cm from the lateral margin in the sagittal plane, the mean IOP and IORP were 25.4 ± 5.2 mmHg and 14 ± 9.2 mmH_2_O, respectively. The IORP satisfactorily reflected the IOP (Pearson correlation coefficient = 0.824, *p* < 0.001).

**Conclusion:**

We measured IOP and IORP simultaneously during orbital compression to gain basic information on pressure changes. In clinical cases, the change in the IOP could be conveniently and noninvasively monitored using continuous IORP measurements.

## Background

Recent studies have focused on the transorbital approach (TOA) as a minimally invasive approach for skull base lesions in the frontal and middle cranial fossa. TOA has several advantages: lesser wounds, minimal brain retraction, reduced cerebrospinal fluid (CSF) leakage, and shorter and less time-consuming access to skull base lesions [1].

However, to date, the TOA has been applied in a limited number of clinical cases, and little is known about its side effects. The possible ophthalmic complications of TOA surgery include enophthalmos, epiphora, ptosis, and diplopia [2]. In addition, many intraorbital complications may arise from direct injury during surgery. Several factors, such as direct cranial nerve injury or extraocular muscle injury due to surgery, may contribute to ocular abnormalities [3]. These complications can also occur when the intraocular pressure (IOP) and intraorbital pressure (IORP) increase due to the increased physical traction by retraction of the orbit and venous pressure during surgery [4–7]. This can lead to visual loss. When the IOP is either momentarily too high or low, it may incur permanent damage to the optic nerve or cause retinal detachment [8]. Therefore, testing the IOP and IORP is critical in TOA surgical patients, and their accurate measurement is essential.

The continuous measurement of IOP and IORP with a retracted orbit remains challenging, despite several measurement methods. We investigated the changes in the IOP and IORP in TOA surgery. Furthermore, we propose a useful measurement method for continuous intraoperative IORP monitoring.

## Methods

This prospective study included consecutive patients who underwent transorbital endoscopic operations from January 2017 to December 2019. Cadaver studies were performed in the Surgical Neuroanatomy Laboratory. The study was approved by the Institutional Review Board and conducted in accordance with the ethical guidelines of the Declaration of Helsinki. Written informed consent was provided by all patients.

### Cadaver studies

Five cadaveric specimens (10 eyeballs) without eye damage, including orbital disease, periorbital disease, and head trauma, were examined. Cadavers with enophthalmos due to premortem or postmortem dehydration were excluded. Cadaveric specimens were processed based on previously described methods [9]. The retractor blade was withdrawn from 0.0 to 2.5 cm in 0.5 cm increments for orbital retraction from the orbital rim to the coronal plane. The average values of three IOP and IORP measurements were obtained.

IOP was measured by a handheld tonometer (Tono-Pen AVIA® Handheld Tonometer, Reichert Inc., USA) and direct cannulation to the eyeball using a Philips IntelliVue MP30 monitor (Philips Medical Systems, Eindhoven, The Netherlands).

This cannulation method consisted of the insertion of a 26-gauge needle into the anterior chamber through the limbus (superomedial position) of each eye [10, 11]. The cannulation was performed based on manufacturer’s instructions and as previously described [12].

The Tono-Pen AVIA® was used very carefully, without applying any orbital pressure, and calibrated according to the manufacturer’s manual. The IOP was effectively measured once a right-angle contact with the corneal surface was established. The average value was recorded three times.

The IORP was measured using a cuff manometer (Cufflator, JT Posey Company, Arcadia, CA, USA) and a 3-mm tracheal tube by placing a pressure measuring cuff on the tracheal tube between the retractor blade and the periorbit.

### Clinical cases

The inclusion criteria were as follows: (1) TOA surgery to treat intracranial pathology, (2) written informed consent from the patient, and (3) no CSF leakage prior to IOP and IORP measurements. The exclusion criteria were as follows: (1) preoperative visual field defect and visual acuity less than 0.05, (2) IOP greater than 20 mmHg preoperatively, and (3) any ophthalmological disease, particularly glaucoma. All patients were tested for IOP, visual acuity, and visual field before and after surgery by the ophthalmologist.

After induction of general anesthesia and intubation, the patient was positioned in an intraoperative supine state with the reverse Trendelenburg at 30 degrees. An oculoplastic surgeon performed the TOA protocol, as per previous cadaver studies [9]. An ophthalmologist measured the IOP at the beginning of the surgery. Pressure measurements were taken at 10-min intervals. A skilled inspector of each measuring instrument, blinded to the results of other tools, measured the parameters.

IOP and IORP were individually measured according to the degree of orbital retraction during surgery. During orbital retraction from the orbital rim to the coronal plane, the retractor blade (DORO® Blades for DORO LUNA® Retractor System) was withdrawn from 0.0 to 2.5 cm in 0.5-cm increments, and an average value was obtained after measuring the IOP and IORP three times.

**Fig. 1:**
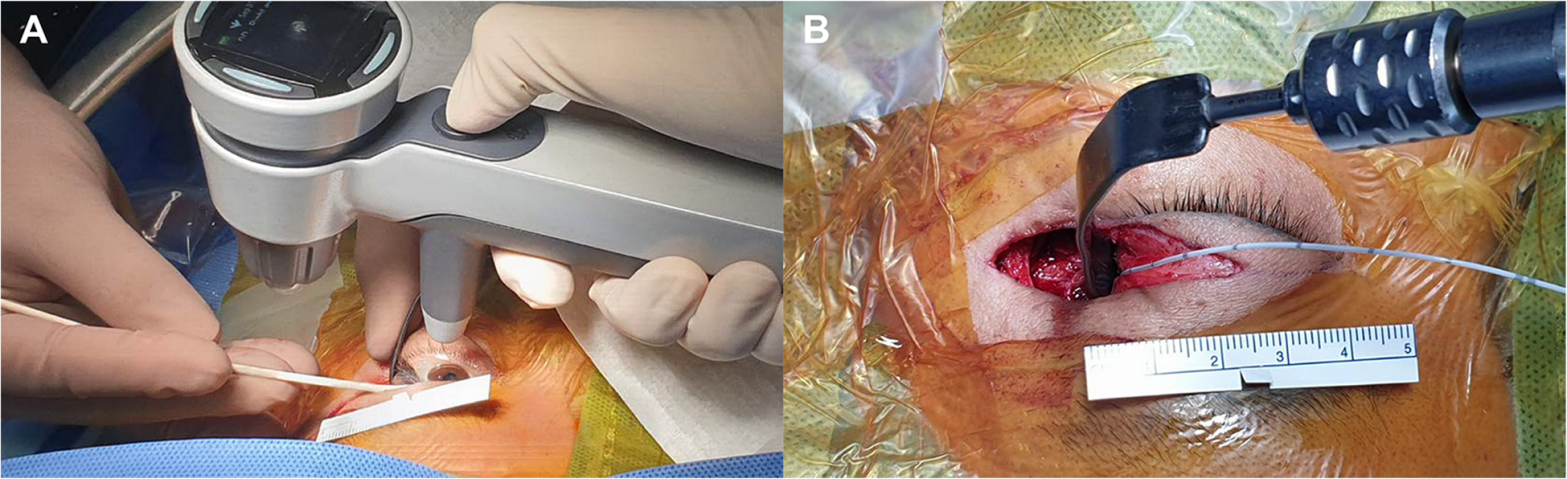
Pressure measurement in clinical cases. **a** IOP measurement by iCare pro tonometer. **b** IORP measurement by micro strain gauge monitor. IOP-intraocular pressure; IORP- intraorbital pressure

The iCare pro (iCare Finland Oy, Helsinki, Finland) tonometer was used to measure the IOP. Similar to the cadaver studies, the average of three final measurements was acquired (Fig. 1a).

A micro strain gauge monitor system (CODMAN MICROSENSOR Transducer & CODMAN® MICROSENSOR® Basic Kit, Codman, Raynham, MA, USA) was used for measuring IORP in clinical cases based on the manufacturer’s instructions (Fig. 1b). The distal catheter tip was placed between the periorbit and the retraction blade.

### Statistical analysis

A paired Student’s t-test was used for the statistical analysis of IOP and IORP changes. A Pearson correlation coefficient was calculated with a linear regression model to assess the correlation between IOP and IORP. SPSS version 22.0 (Statistical Package for Social Sciences, SPSS Inc., Chicago, IL, USA) was employed for statistical analyses, with a significance level set to *p* < 0.05.

## Results

### Cadaver studies

**Fig. 2:**
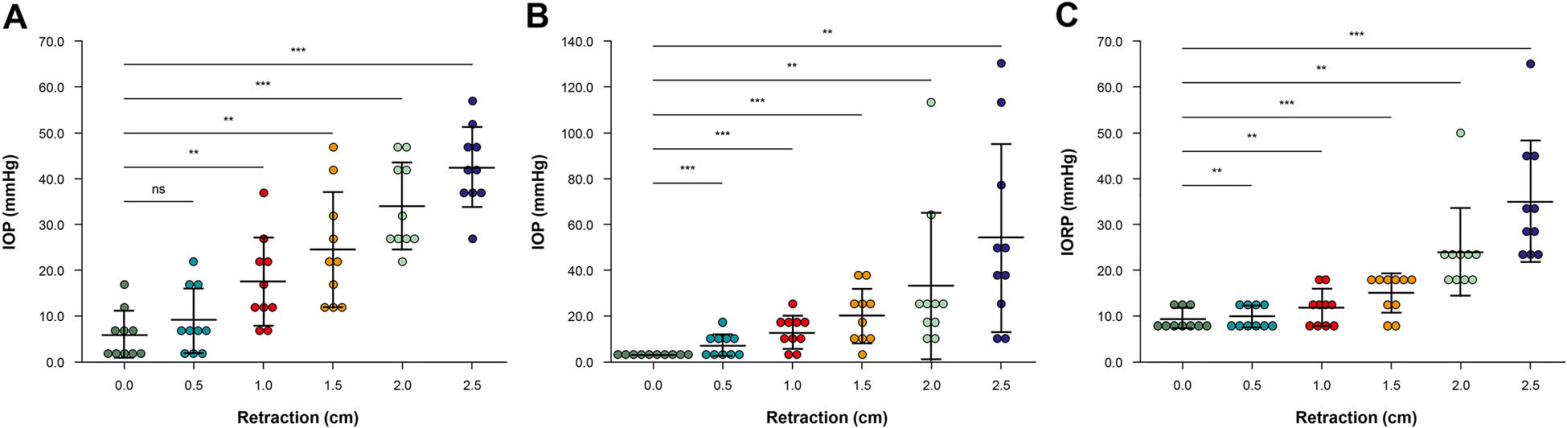
Pressure measurement in cadaveric study. **a** IOP measured by Tono-Pen AVIA®. **b** IOP measured by cannula needle monitor. **c** IORP measured by manometer. ns = not significant; ^*^p < 0.01; ^**^p < 0.001. IOP-intraocular pressure; IORP- intraorbital pressure

The mean IOP ± SD values measured by Tono-Pen AVIA® at every 0.5-cm retraction, from 0.0 to 2.5 cm, were 4.9 ± 5.78, 8.2 ± 6.92, 17.2 ± 9.59, 24.1 ± 12.08, 34.1 ± 10.32, and 41.9 ± 9.04 mmHg. A greater degree of retraction increased the IOP proportionately (Fig. 2a).

The IOP, as measured by the cannula needle, was set to 0 mmHg prior to orbital retraction. The mean IOP ± SD values measured by the cannula pressure monitoring method were 0.0, 6.6 ± 3.72, 12.8 ± 6.58, 19.5 ± 10.75, 32.9 ± 32.00, and 54.6 ± 40.49 mmHg at each retraction (Fig. 2b).

The mean IORP±SD values measured by the cuff manometer were 8.5 ± 2.55, 10.1 ± 2.33, 12.4 ± 3.44, 15.3 ± 4.19, 24.4 ± 9.44, and 35.2 ± 12.99 cmH2O, when the orbit was retracted from 0.0 to 2.5 cm at an interval of 0.5 cm (Fig. 2c).

### Patient characteristics

The demographic data of clinical subjects and their IOP are presented in Table 1. Of the nine patients, one was male. The mean age was 44.5 ± 14.3 years (range, 23–70 years). The pathological findings included schwannomas (*n* = 5), meningiomas (*n* = 3), and glioma (*n* = 1). The sites of the tumor differed, but all were accessible via TOA surgery. The mean tumor size (±SD, range) was 26.6 mm (±7.4, 20–41.7, maximum diameter). The mean preoperative and postoperative IOP values (±SD, range) were 13.2 mmHg (±1.7, 11–16) and 13.0 mmHg (±1.3, 11–14) on the left side, and 13.3 mmHg (±1.9, 11–16) and 14.3 mmHg (±2.6, 11–19) on the right side, respectively. There was no difference in visual acuity or visual field before and after the operation.

**Table 1:** Patient data in clinical cases

No.	Sex	Age	Pathology	Location	Tumor size (mm)	Surgical position, head position	Preop. IOP		Postop. IOP	
							Lt	Rt	Lt	Rt
1	F	39	Schwannoma	V2, PPF, Lt.	12.5 × 23.4 × 24.4	Supine, neutral	14	16	14	19
2	F	42	Schwannoma	Trigeminal, Lt.	21 × 18.8 × 23.2	Supine, neutral	16	15	14	16
3	F	42	Meningioma	CS wall, Lt.	31.1 × 32.6 × 35.2	Supine, neutral	15	12	11	12
4	F	42	Meningioma	Temporal tip, Lt.	17.5 × 20.3 × 19.5	Supine, neutral	11	14	12	13
5	F	47	Schwannoma	Trigeminal, Rt.	30 × 21.4 × 24.9	Supine, neutral	12	11	13	13
6	F	35	Schwannoma	Trigeminal, Rt	41.7 × 31.5 × 27.3	Supine, neutral	11	12	11	13
7	F	70	Meningioma	ACP, Lt.	28 × 19.1 × 22.2	Supine, neutral	14	15	14	17
8	M	58	Schwannoma	CS, Rt.	25.1 × 23 × 22.4	Supine, neutral	13	14	14	15
9	F	23	Glioma	Temporal tip	20.3 × 21.2 × 18.6	Supine, neutral	13	11	14	11

*ACP* anterior clinoid process, *CS* cavernous sinus, *F* female, *IOP* intraocular pressure, *Lt* left; *M* male, *Postop.* postoperative, *PPF* pterygopalatine fossa, *Preop.* preoperative, *Rt* right

### IOP and IORP in clinical cases

**Fig. 3:**
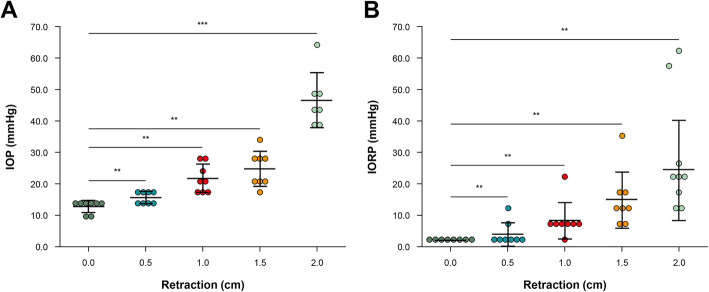
Pressure measurement in clinical cases. **a** IOP measured by tonometry. **b** IORP measured by micro strain gauge manometer. ns = not significant; ^*^p < 0.01; ^**^p < 0.001. IOP-intraocular pressure; IORP- intraorbital pressure

**Fig. 4:**
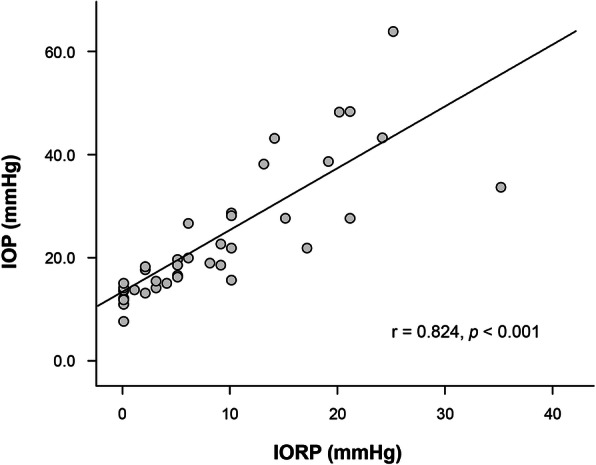
Linear regressions for IOP and IORP according to orbit retraction in clinical cases. r = Pearson correlation coefficient. IOP-intraocular pressure; IORP- intraorbital pressure

The initial mean value of intraoperative IOP of the tumor side measured by iCare was 12.7 ± 2.33 mmHg. The mean IOP values consecutively measured by increasing the distance of the retraction blade from 0.0 to 2.0 cm, at an interval of 0.5 cm from the lateral orbital wall, were 12.7 mmHg (±2.3, 8–15), 15.8 mmHg (±1.8, 13.5–18.6), 21.1 mmHg (±4.5, 15.8–28), 25.4 mmHg (±5.2, 18.9–34), and 46.6 mmHg (±8.8, 38.5–64.2), forming a curvilinear pattern (Fig. 3a). The IORP was continuously measured using a micro strain gauge monitor, increasing the retraction by 0.5 cm from 0.0 to 2.0 cm. The mean IORP values were 0.0 mmHg, 3.5 mmHg (±2.9, 1–10), 7.8 mmHg (±5.7, 3–21), 14 mmHg (±9.2, 6–35), and 24.8 mmHg (±15.6, 13–62). As the retraction increased, the IORP also increased in a curvilinear pattern (Fig. 3b). The pressure increased gradually when the retraction was less than 1.5 cm, but increased abruptly when the retraction was greater than 1.5 cm. Finally, the mean IOP and IORP values increased to 46.6 mmHg from 12.7 mmHg and to 24.8 mmHg from 0.0 mmHg, respectively, when the orbit was compressed to 2.0 cm. In a linear regression analysis of the differences between IOP and IORP, there was a significant correlation between IOP and IORP (Pearson correlation coefficient = 0.824, *p* < 0.001) (Fig. 4).

## Discussion

### Risk of increased IOP and IORP

The IOP indicates a dynamic balance between the formation and outflow of aqueous humor [13]. The mean IOP is approximately 14–16 mmHg (±3.5 mmHg during a 24-h cycle) in the general population, and an IOP between 7 and 21 mmHg is considered normal [13–15].

A normal IORP ranges from 3 to 6 mmHg [16, 17]. A rapid increase in IORP is associated with orbital structural damage causing irreversible vision loss [18]. The proposed mechanism underlying this ophthalmologic complication is a rapid increase in IORP within the rigid confines of the orbit, resulting in hypoperfusion of critical neural structures. This is defined as orbital compartment syndrome (OCS). In OCS, optic nerve and retinal damage may develop into rapidly irreversible vision loss.

An elevated IOP due to orbit retraction during TOA surgery might lower the eye perfusion pressure, leading to perioperative visual loss (POVL). High IOP is still the most important risk factor linked to an untreated glaucomatous eye (> 21 mmHg) progressing to a severe stage. Glaucoma is characterized by optic nerve atrophy and impaired vision, and is the main cause of increased IOP causing neuropathy [19–22].

There are no reports of OCS complications in the application of TOA, even if these are likely to occur. Although no serious adverse events such as temporary or permanent POVL or visual disturbance due to an increase in IOP during surgery are reported in TOA surgery, neurosurgeons should be aware and cautious of the risks.

### IOP and IORP measurements

Currently, the most accurate clinical tonometer is the Goldmann applanation tonometer (GAT). When measuring IOP, iCare yields high values compared to the GAT, but the results are similar enough for iCare to be used as an alternative when it is difficult to use the GAT due to its high correlation [23]. In this study’s clinical cases, the GAT was replaced with iCare for its ease of measuring the intraoperative IOP.

In the past, the slit catheter technique, direct orbital manometry, and orbitonometer methods were used for measuring IORP [16, 17, 24]. In this study, we used a micro strain gauge as a less invasive method and found it to be useful.

### Surgical considerations

An increased orbital pressure for over 60–100 min can cause permanent visual complications [18, 25]. Although there may be differences depending on the applied pressure, position, and contact area, the mean recovery time of IOP was reported to be 22.3 ± 2.2 min when a 15-g weight was applied for 1 min [26]. Therefore, it is necessary to consciously relieve the pressure on the orbit during TOA surgery. We collected basic information on the possible retraction range that would not cause complications during TOA surgery.

We intended to validate an instrument for measuring IOP and IORP and quantify pressure increments due to external ocular compression during TOA surgery. Our results showed that as the orbit was retracted, IOP and IORP increased in a curvilinear pattern (Fig. 3). In the cadaver study, we found that both IOP and IORP increased rapidly when pulled over 2 cm on inspection using a cuff manometer and a cannula needle monitoring system (Fig. 2). The slope of the ascending curve of iCare measurements showed a gradual increase in IOP when retracting the orbit to less than 1.5 cm, and an abrupt rise above 1.5 cm. Furthermore, when the orbit was compressed to 2.0 cm during TOA surgery, the mean IOP was 46.6 ± 8.8 mmHg, with the highest value being 64.2 mmHg (range, 38.5–64.2 mmHg).

The preferred method for measuring IOP is iCare tonometry through direct contact with the cornea. If measured during TOA surgery, it is impossible to detect an increased IOP induced by the excessive traction of the orbit continuously. However, IORP could be measured continuously using a micro strain gauge monitor system. In addition, IORP measured using a micro strain gauge shows a high correlation with IOP measured using the iCare tonometer (Fig. 4). This IORP measurement method could be useful in compensating for the shortcomings of the tonometer.

### Limitations

There are some limitations to this study. First, only a small number of patients who had a normal IOP were investigated. Second, we did not consider the geometry of the retractor and contact surface. Third, since the micro gauge catheter tip has a very small contact area, the IORP may not be accurately reflected based on the position of the catheter tip. Finally, we did not consider changes in IOP due to delayed surgical time, damage of the periorbita, or CSF leakage, and did not compare the blood pressure or the reverse Trendelenburg level at the time of IOP measurement. We are in the process of data collection, and these limitations will be addressed in future studies.

## Conclusions

We measured IOP and IORP simultaneously in cadaver and clinical cases of transorbital surgery. The IOP and IORP increase in a curvilinear pattern when the orbit is retracted. Retracting the orbit medially to more than 1.5 cm from the lateral orbital wall may increase IOP to a critical level. The change in IOP can be conveniently and noninvasively monitored using continuous IORP measurements.

## Data Availability

The datasets generated during and/or analysed during the current study are available from the corresponding author on reasonable request.

## Abbreviations

CSF: Cerebrospinal fluid
GAT: Goldmann applanation tonometer
IOP: Intraocular pressure
IORP: Intraorbital pressure
TOA: Transorbital approach
OCS: Orbital compartment syndrome
POVL: Perioperative visual loss
SevEN: Severance Endoscopic Neurosurgery study group
